# An ecological study of freshwater ecosystem and its colligation to Odonates assemblages in Ipogun, Southwest Nigeria

**DOI:** 10.1186/s42269-022-00774-4

**Published:** 2022-04-01

**Authors:** Babasola Adu, Omolola Dada, Victor Tunwase

**Affiliations:** Department of Biology, The Federal University of Technology Akure, Akure, Ondo State Nigeria

**Keywords:** Odonata, Diversity, Alaasin, Idi and Aponmu

## Abstract

**Background:**

Odonata (dragonfly and damselfly) are particularly good indicators of freshwater ecosystem health. The constant disturbance of freshwater habitats can result in the reduction of Odonata species diversity. Changes in Odonata biodiversity are influenced by several human activities, such as agriculture, urbanization, input of pollutants in water and construction. This study was carried out to assess the abundance and diversity of Odonata, evaluate the physicochemical characteristics of water, and compare the community structure of Odonata at three selected sites along River Aponmu in Ipogun. Adult odonates were sampled and identified for 11 months using a sweep net, water samples were collected and some parameters were determined during the study period.

**Results:**

A total of 906 specimens representing sixty-four (64) species and sixteen (16) genera in seven (7) families (Coenagrionidae, Lestidae, Platycnemididae, Chlorocyphidae, Calopterygidae, Libellulidae, and Gomphidae) were collected and identified. Of the 906 specimens, Libellulidae had the highest percentage composition (44%) with 395 individuals out of which *Trithemis arteriosa* (a pollution tolerant species) had the highest number of individuals (225) and Gomphidae had the lowest percentage composition (0.03%) with 1 individual. Most of the species collected are known for their tolerance to disturbed environments. They include *Pseudagrion melanicterum*, *Paragomphus genei,* and *Orthetrum Julia*. Aponmu area had the highest species diversity (*H*′ = 2.312) while Idi area had the least species diversity (*H*′ = 2.021). Alaasin area had the highest Simpson_*d* value (0.8557) and the best taxa distribution (Evenness = 0.524; Equitability_*J* = 0.7764) which makes the area more pristine than other sites while Aponmu area had the least distribution (Evenness = 0.3365; Equitability_*J* = 0.6798). Analysis of variance (ANOVA) result of physicochemical parameters revealed that temperature (°C), pH, Dissolved Oxygen (mg/L), turbidity (NTU), Biochemical Oxygen Demand (mg/L), NO_3_ (mg/L), and PO_4_ (mg/L) did not show significant difference at the three sites while EC (µS/cm) and TDS (mg/L) which have moderately high mean values indicated significant difference at Aponmu area (*p* < 0.05). *T. arteriosa* exhibited a weak negative correlation to both temperature and DO.

**Conclusions:**

This study has provided information on Odonata assemblage at River Aponmu and infers based on the assemblage that the river may be somewhat polluted at the period the research was carried out. It is therefore recommended that efforts should therefore be taken to discourage water pollution in order to preserve the diversity of these insects and the water quality.

## Background

Aquatic insects represent an integral part of freshwater ecosystems and they contribute to various ecological services in the ecosystem. The use of aquatic insects such as dragonflies and damselflies (Odonata) as indicators of water quality and health is very important in determining ecological integrity of freshwater ecosystem. This is largely based on their tolerance level, as some cannot tolerate mild pollution; some have moderate tolerance for pollution, while others may thrive even in heavily polluted environment (Arimoro and Ikomi 2008; Adu et al. 2016a). The abundance and diversity of these insects depict the health status of the aquatic environment (Kehinde et al. 2014). The insect order has two sub-orders (Anisoptera and Zygoptera) with about 6500 species (Dijkstra 2007). Odonata as aquatic insects has its larva stage living in water and adult stage as terrestrial insects. This positioned them as a powerful assessment tool for both aquatic and terrestrial environments (Oertli 2008). They are able to indicate habitat degradation due to their sensitivity to changes in habitat structure (Clausnitzer 2003; Clark and Samways 1996). The extent of degradation in a particular freshwater habitat can be determined by the species of Dragonflies and Damselflies found there (Samways 1993). Odonates are specifically useful as habitat quality indicators as different species are often restricted to different sites. Therefore changes in species composition in a community may imply environmental disturbance, which alter physical, chemical, and biological characteristics of the water body (Stewart and Samways 1998). Changes in physico-chemical characteristics such as pH, dissolved oxygen, temperature, and electrical conductivity may affect the community structure of Odonata (Corbet 1999; Remsburg and Turner 2009). Some physicochemical parameters have been found to affect the diversity of Odonata. Water temperature influences the type and number of species present in a place since they require optimum temperature to live, thrive, lay eggs and emerge into adults (Dallas and Ross-Gillespie 2015). Popoola and Otalekor (2011), found that there was more species diversity at a relatively lower temperature than the result got when there was corresponding rise in temperature. Dissolved Oxygen (DO) is major parameter for assessing the health of the water bodies and according to Kemabonta et al. (2020), maybe inversely related to diversity.

Odonata predatory ability makes them useful in biological control of disease vectors such as mosquitoes and freshwater snails and their involvement in biomonitoring of aquatic ecosystems is attributed to their sensitivity to human disturbance (Knight et al. 2005). Regular monitoring of Odonata assemblages may help to determine the effect of human disturbance on water quality (Suhling et al. 2009). Owing to the usefulness of Dragonflies and Damselflies in ecological studies, the assessment of their presence, distribution, diversity, and abundance in and around freshwater bodies is of paramount importance. The study aimed at determining the physico-chemical characteristics and Odonata community structure of River Aponmu, Ipogun Southwest Nigeria, and the colligation of the Odonate diversity to the health status of the River.

## Methods

### Study area

The study was carried out at Ipogun village, Ifedore local government Area of Ondo State, Nigeria. Ipogun (7° 18′ 53″ North 5° 04′ 48″ East) is a rural village adjacent Ilara-Mokin in Ondo State, Nigeria with an estimated population of about 6000. It is about 14 km from Akure the state capital of Ondo State. The climate in Ipogun is tropical with wet and dry seasons. The wet season (March–October) is characterized by heavy rains with occasional flooding of river banks and a dry season (November–February) distinguished by increased temperature, very little or no rainfall and consequently, the river dries up with a few stagnant pockets of water along its course. The inhabitants of the village are largely engaged in farming. The primary source of water supply for agricultural and domestic activities is the ‘Aponmu’ river, flowing through the village. The study was carried out at River Aponmu.

### Study sites

**Fig. 1:**
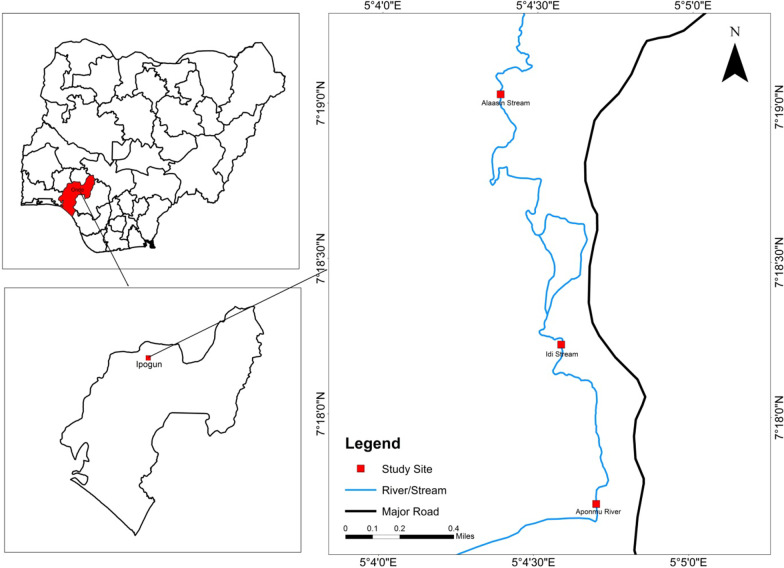
Map of study area showing the study sites in Ipogun, Ifedore Local Government, Ondo State

Three study sites (Alaasin, Idi, and Aponmu) were selected along the course of River Aponmu (Fig. 1). The study sites were chosen based on the surrounding vegetation type and different human activities around the water bodies. The study sites are all part of River Aponmu but given different names by the people of the sites.

### Alaasin stream

Alaasin stream (7° 19′ 2″ N 5° 4′ 23″ E) is a flowing stream that passes through cocoa, plantain, and banana farms. The stream is characterized by rocky beds; boulders can be seen at some part of the bank of the stream, while the bottom substrate is mainly silt. It is a permanent stream, though the water level reduces greatly during dry season but does not dry up completely. Other vegetation at the site includes grasses and shrubs. The cocoa trees, plantain plantation, and oil palm trees form shade at some part of the site. Human activities carried out at the site include washing of motorcycles, clothes, and farming.

### Idi stream

Idi stream (7° 18′ 14″ N 5° 4′ 42″ E) is a slow-flowing water body and shallow, especially across major footpaths leading to Cocoa farms. “Idi” is a native name given to the area by the people living there. The surrounding of the stream is characterized by the presence of crop trees (e.g. cocoa trees and palm trees) and some shrubs. The site is shaded by Cocoa trees and a few other trees. The stream bed is made up of boulders, sand-loam soil, and rocks. The water is clear and sometimes dries up during dry season. The stream is influenced by regular cutting of surrounding vegetation and exhaust fumes from passing automobiles.

### River Aponmu

River Aponmu (7° 17′ 44″ N 5° 4′ 49″ E) is an open river which allows direct penetration of sunlight. The river bed is made up of sand-loam soil, boulders, and stone. The water is moderately clear and slow-flowing. The major types of vegetation around the river are grasses and shrubs. Human activities here include regular cutting of riparian vegetation, construction work, and laundry. Other activities at the site include cassava processing mill. Some patches of human excrement can be seen in some parts of the site.

### Sample collection

The research was carried out from January 2020 to December 2020 (with the exception of April 2020 due to the strict lockdown enforced as a measure against the spread of COVID-19 pandemic). The sampling period comprises of 4 months of dry season and 7 months of wet season. All samples were collected once a month between the hours of 9:00 a.m. and 2:00 p.m.

### Sampling, identification, and preservation of Odonate specimens

Only adult specimens were sampled under favorable weather condition between the hours of 9:00 a.m. and 2:00 p.m. The adults were sampled using a large white sweep net with an orifice of 72 cm. The length of the net was long enough to be able to fold close so that the specimen(s) caught would not escape when flipping the rim over the net. When a dragonfly or damselfly was seen flying around, the net was swung from behind to capture it. Specimens that were difficult to identify at the field were taken to the laboratory for proper identification using standard identification manual: Dijkstra and Clausnitzer (2014). The specimens were also identified with Odonata pictures on the African Dragonfly and Damselfly Database (ADDO). The specimens collected were placed inside well-labeled white triangular envelopes at the site. The wings were carefully folded at the back to prevent it from getting rumpled. Male and female caught in tandem were kept in the same envelope. All specimens collected were soaked in acetone for minimum of 12 h to preserve it and retain the beautiful pigment coloration of the specimens. The samples were then removed from acetone and air-dried by placing them on tissue paper on the work table in the laboratory. The dried specimens were thereafter placed again in white rectangular envelope and placed in a well-labeled white box for future references.

### Water sampling and analysis (physicochemical parameters)

Collection of water samples and measurement of some physicochemical parameters were done concurrently for 11 months. Water samples were collected using 1 L plastic bottles from the three study sites for analysis. Before collection of the water, the bottles were thoroughly rinsed with the stream water to be collected and the one for analysis was later collected inside the bottle. The sample bottles were labeled to indicate the site, name of the site, water type, date collected, and sample source. Sampling was done by lowering pre-cleaned plastic bottles into the water at accessible points. The plastic bottles were lowered to a depth of about 30 cm and allowed to overflow before withdrawing. A total of 33 water samples were analyzed throughout the sampling period. Some water parameters were measured in situ. They include Ambient and Water temperature (°C), pH, Total dissolved solid (mg/L), Dissolved oxygen (mg/L), Electrical conductivity (µS/cm) using HANNA multimeters. Water Turbidity (NTU) was also determined using turbidity meter (WGZ-B, China). Biochemical oxygen demand (BOD) was determined using Winkler’s method, nitrate and phosphate were determined in the laboratory using the HACH DR 2000 direct reading spectrophotometer, method 8039 and 8048 respectively. The HACH nitraVer 5 powder pillow reagents were added to 25 ml of water sample against deionized water at a wavelength of 500 nm for nitrate while The HACH phosVer 3, phosphate powder pillow reagent was added to 25 ml of the water sample against deionized water at a wavelength of 890 nm for phosphate.

### Statistical analysis

All data collected from the three study sites for the 11 months period were accumulated and analyzed statistically using inferential and descriptive statistics. Data on physicochemical parameters were subjected to one-way analysis of variance (ANOVA) (*p* < 0.05), and were significant difference existed, means were separated using the Tukey’s test. ANOVA was done using SPSS 23.0 software package. Pearson correlation analysis was done to determine the relationship between physicochemical parameters and Odonata. Data on taxonomic composition and diversity of Odonata species at the three study sites (Alaasin, Idi, and River Aponmu) in Ipogun were subjected to diversity indices such as Shannon-Weiner (*H*′), Simpson 1-D, Margalef, Evenness (*E*) and Equitability using Paleontologist Statistical Software package (PAST) version 3.0. All the graphs were plotted using Microsoft Excel 2016.

## Results

### Abundance of Odonata at the study sites

**Fig. 2:**
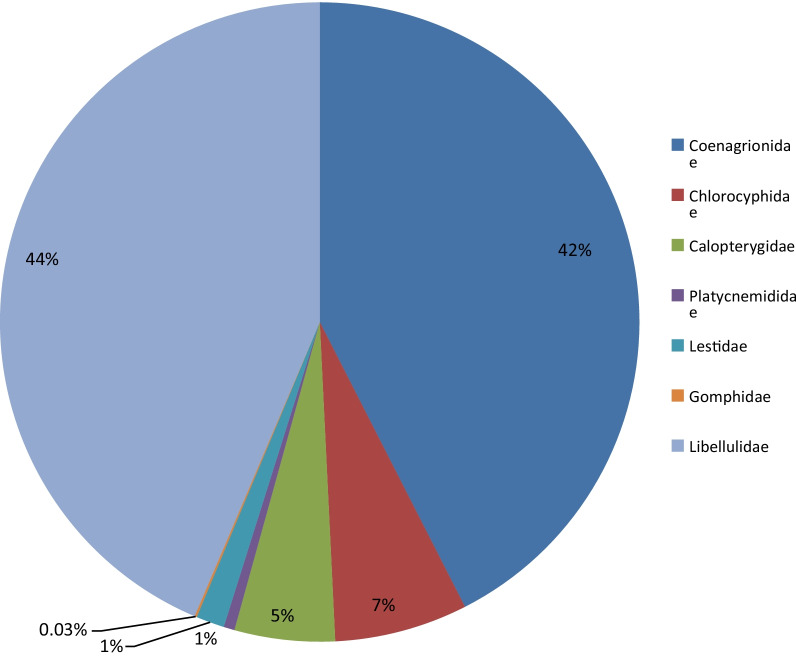
Percentage composition of the seven Odonata families collected from three sites at Ipogun in 2020

A total of 906 adult individual odonates were collected from the three study sites out of which 510 (56%) were damselflies while 396 (44%) were dragonflies. A total of 320 individuals were collected from Alaasin stream, 163 from Idi stream and 423 from River Aponmu respectively. Eighteen (18) different species belonging to five (5) families were recorded at Alaasin stream. The families include: Coenagrionidae, Chlorocyphidae, Calopterygidae, Platycnemididae and Libellulidae. Sixteen (16) species belonging to five (5) families were recorded at Idi stream. The families include Coenagrionidae, Chlorocyphidae, Calopterygidae, Platycnemididae, and Libellulidae. At river Aponmu, thirty (30) different species belonging to seven (7) families were recorded. These families include Coenagrionidae, Lestidae, Platycnemididae, Chlorocyphidae, Calopterygidae, Libellulidae, and Gomphidae. River Aponmu had the highest number of species while the least number of species was recorded at Idi stream. *Pseudagrion melanicterum* was the dominant species collected at Alaasin stream (76) and Idi stream (57) while *Trithemis arteriosa* (144) was dominant at river Aponmu. Figure 2 shows the percentage composition of families of Odonata in the three study sites with Libellulidae having the highest percentage (44%), next is Coenagrionidae (42%) and Gomphidae with the least percentage (0.03%). Table 1 reveals the checklist of Odonata collected from three sites along the course of River Aponmu in Ipogun.

**Table 1:** Checklist of Odonata collected from three sites along River Aponmu in Ipogun

Family	Taxa	Numbers collected			Total
		Alaasin	Idi	Aponmu	
Coenagrionidae	*Pseudagrion kersteni* (Gerstäcker, 1869)	64	28	91	183
	*Pseudagrion melanicterum* Selys, 1876	76	57	28	161
	*Pseudagrion nubicum* Selys, 1876	4	0	0	4
	*Pseudagrion hamoni* Fraser, 1955	2	0	0	2
	*Pseudagrion sublacteum* (Karsch, 1893)	0	1	2	3
	*Pseudagrion glaucum* (Sjöstedt, 1900)	0	0	2	2
	*Pseudagrion torridum* Selys, 1876	0	0	1	1
	*Pseudagrion sudanicum* Le Roi, 1915	0	0	3	3
	*Africallagama vaginale* (Sjöstedt, 1917)	5	0	1	6
	*Africallagama subtile* (Ris, 1921)	4	1	5	10
	*Agriocnemis maclachlani* Selys, 1877	1	0	0	1
	*Agriocnemis victoria* Fraser, 1928	0	1	2	3
	*Ceriagrion glabrum* (Burmeister, 1839)	0	0	6	6
Chlorocyphidae	*Chlorocypha curta* (Hagen in Selys, 1853)	19	6	2	27
	*Chlorocypha glauca* (Selys, 1879)	19	3	7	29
	*Chlorocypha pyriformosa* Fraser, 1947	4	1	0	5
Calopterygidae	*Sapho ciliata* (Fabricius, 1781)	2	0	0	2
	*Phaon iridipennis* (Burmeister, 1839)	12	0	18	30
	*Phaon camerunensis* Sjöstedt, 1900	0	0	2	2
	*Umma cincta* (Hagen in Selys, 1853)	4	8	0	12
Platycnemididae	*Mesocnemis singularis* Karsch, 1891	2	0	1	3
	*Elattoneura nigra* (Kimmins, 1938)	0	1	1	2
Lestidae	*Lestes pinheyi* Fraser, 1955	0	0	4	4
	*Lestes pallidus* Rambur, 1842	0	0	9	9
Gomphidae	*Paragomphus genei* (Selys, 1841)	0	0	1	1
Libellulidae	*Trithemis arteriosa* (Burmeister, 1839)	55	26	144	225
	*Trithemis imitata* Pinhey, 1961	0	1	2	3
	*Trithemis furva* Karsch, 1899	0	0	1	1
	*Trithemis aconita* Lieftinck, 1969	0	0	1	1
	*Palpopleura lucia* (Drury, 1773)	5	9	12	26
	*Palpoleura portia* (Drury, 1773)	0	12	0	12
	*Palpoleura jucunda* Rambur, 1842	0	2	0	2
	*Palpopleura deceptor* (Calvert, 1899)	0	0	5	5
	*Brachythemis wilsoni* Pinhey, 1952	21	0	26	47
	*Orthetrum julia* Kirby, 1900	21	6	16	43
	*Orthetrum stemmale* (Burmeister, 1839)	0	0	14	14
	*Orthetrum guineense* Ris, 1910	0	0	4	4
	*Orthetrum chrysostigma* (Burmeister, 1839)	0	0	12	12
Total		320	163	423	906

Table 2 shows the diversity indices of Odonata adults collected during the study period. River Aponmu had the highest number of species (30) and individuals (423) and is also the most diverse in terms of species richness (*H*′ = 2.312). Species distribution at the site is the lowest among the three sites with Evenness value of *E*: 0.3365 and Equitability of 0.6798. All other diversity indices used at the site were high (Margalef 4.795; Simpson *d*: 0.8221) when compared to Alaasin and Idi. Idi stream accounted for the least number of species (16) and the least number of Individuals (163). Shannon Wiener diversity index (*H*′ = 2.021) revealed that the site is the poorest in terms of species diversity. However, Evenness value revealed that Idi stream (*E*: 0.4716) is better species distribution than River Aponmu (*E*: 0.3365). Of the three sites studied, Idi stream had the least value for Simpson *d* (0.8085) and Equitability (0.7289). Eighteen (18) species of Odonata comprising of three hundred and twenty (320) individuals were represented at Alaasin stream which is better than the number recorded at Idi stream. Shannon Wiener index (*H*′ = 2.244) at this site is not the highest of the three sites but is higher than that of Idi stream (*H*′ = 2.021). Although Alaasin stream had a low Margalef value (2.947) but had the highest Evenness (*E* = 0.524) and Equitability value (0.7764) when compared to the other sites. This shows that the distribution of species of Odonata at this site is the best. Alaasin stream also had the highest value for Simpson *d* (0.8557) which depicts a higher level of dominance than the two other sites.

**Table 2:** Diversity indices of Odonata collected from three sites along River Aponmu in Ipogun

Diversity indices	Alaasin	Idi	Aponmu
Taxa_S	18	16	30
Individuals	320	163	423
Simpson (1-D)	0.8557	0.8085	0.8221
Shannon (*H*′)	2.244	2.021	2.312
Margalef	2.947	2.945	4.795
Equitability_*J*	0.7764	0.7289	0.6798
Evenness (*E*)	0.524	0.4716	0.3365

### Physicochemical parameters

Mean and Standard Error of the physicochemical parameters of the three sites during the study period (Table 3) shows that the water temperature, pH, ambient temperature, and Turbidity at the sites are not significantly different from each other. The conductivity value at Aponmu (178.16 ± 8.62) is significantly different from the values for Alaasin (135.27 ± 0.33) and Idi (147.73 ± 5.73) which are not significantly different from each other. The mean and standard error values for dissolved oxygen (DO) and biochemical oxygen demand (BOD) are not significantly different from each other at all the sites. However, the total dissolved solid (TDS) value at Aponmu (162.82 ± 7.83) showed a significant difference from the values at Alaasin (109.77 ± 2.89) and Idi (125.77 ± 3.65). The calculated values for mean and standard error for Phosphate (PO_4_) and Nitrate (NO_3_) at the three study sites are not significantly different from one another.

**Table 3:** Mean and standard error of the physicochemical parameters of three sites in Ipogun

Parameters	Study sites		
	Alaasin	Idi	Aponmu
Ambient temperature (°C)	30.27 ± 0.33^a^	30.36 ± 0.44^a^	30.73 ± 0.34^a^
Water temperature (°C)	26.30 ± 0.32^a^	26.25 ± 0.32^a^	26.51 ± 0.33^a^
Ph	6.97 ± 0.59^a^	6.92 ± 0.05^a^	6.89 ± 0.06^a^
DO (mg/L)	5.77 ± 0.17^a^	5.61 ± 0.14^a^	5.73 ± 0.14^a^
Conductivity (µS/cm)	135.27 ± 4.69^a^	147.73 ± 5.73^a^	178.16 ± 8.62^b^
Turbidity (NTU)	51.18 ± 1.26^a^	48.35 ± 1.21^a^	52.35 ± 1.23^a^
TDS (mg/L)	109.77 ± 2.89^a^	125.77 ± 3.65^a^	162.82 ± 7.83^b^
BOD (mg/L)	3.06 ± 0.14^a^	2.86 ± 0.12^a^	2.96 ± 0.09^a^
PO_4_ (mg/L)	0.17 ± 0.01^a^	0.18 ± 0.01^a^	0.19 ± 0.01^a^
NO_3_ (mg/L)	0.08 ± 0.01^a^	0.09 ± 0.01^a^	0.11 ± 0.01^a^

Mean followed by similar alphabets indicate no significant difference (*p* > 0.05) using Tukey’s test

At Alaasin stream, *Pseudagrion Kersteni* showed a fairly strong negative correlation with both ambient and water temperature which is also significant at *p* < 0.05 (Table 4). There is a strong negative correlation coefficient between *Pseudagrion melanicterum* and water temperature (*p* < 0.01). *P. melanicterum* also revealed a weak negative correlation with turbidity (*p* < 0.01). *Trithemis arteriosa*revealed a moderately strong positive correlation with biochemical oxygen demand (BOD) which is significant at 0.05 levels. *Orthetrum julia* showed no correlation with turbidity and phosphate (PO_4_). The correlation coefficient values of Odonata taxa as influenced by physicochemical parameters at Idi stream is shown in Table 5. *Trithemis arteriosa* had a fairly strong positive correlation with nitrate (NO_3_). The correlation is significant (*p* < 0.05). *Umma cincta* had no correlation with EC and *T. imitata* had a significant strong negative correlation with turbidity and water temperature (*p* < 0.01). At River Aponmu, *P. kersteni* revealed a significant strong positive correlation with DO, EC, and TDS (*p* < 0.05). *Pseudagrion sudanicum* had significant strong positive correlation with DO (*p* < 0.05) and a significant strong negative correlation with BOD (*p* < 0.05). *Paragomphus genei* had no correlation with pH. *T. imitata* had a significant strong positive correlation with NO_3_ (*p* < 0.01). The correlation coefficient values of Odonata taxa with the parameters at river Aponmu are shown in Table 6.

**Table 4:** Correlation coefficient (Pearson *r*) values of Odonata taxa as influenced by physicochemical parameters at Alaasin stream

Odonata	Parameters									
	Amb. temp (°C)	pH	DO (mg/L)	EC (µS/cm)	TURBIDITY (NTU)	WATER TEMP (°C)	TDS (mg/L)	BOD (mg/L)	PO_4_ (mg/L)	NO_3_ (mg/L)
*P. kersteni*	− 0.71*	0.18	0.14	− 0.441	− 0.42	− 0.67*	0.205	0.367	− 0.28	0.18
*P. melanicterum*	− 0.41	0.50	− 0.36	− 0.05	− 0.08**	− 0.91**	0.13	0.46	0.14	0.48
*P. nubicum*	− 0.30	− 0.53	0.42	− 0.20	0.39	0.09	0.52	− 0.58	− 0.41	− 0.50
*P. hamoni*	0.36	− 0.49	− 0.07	0.09	0.01	0.10	0.39	− 0.09	− 0.09	− 0.20
*A. vaginale*	0.35	− 0.53	− 0.13	0.00	0.10	0.11	0.33	− 0.01	− 0.17	− 0.23
*A. subtile*	− 0.24	− 0.22	0.23	− 0.39	0.67*	0.09	0.18	− 0.14	− 0.61*	− 0.47
*A. maclachlani*	− 0.03	0.18	0.54	0.07	0.32	0.10	0.44	− 0.41	− 0.29	− 0.36
*C. curta*	− 0.09	0.19	0.19	− 0.02	− 0.29	− 0.20	0.06	0.07	− 0.12	0.04
*C. glauca*	0.65*	− 0.07	− 0.11	− 0.02	0.07	0.33	− 0.17	0.22	0.44	0.32
*C. pyriformosa*	− 0.03	0.18	0.54	0.07	0.32	0.10	0.44	− 0.41	− 0.29	− 0.36
*S. ciliata*	0.36	0.30	− 0.40	0.31	− 0.02	− 0.02	− 0.26	0.08	0.66*	0.51
*P. irridipennis*	0.18	− 0.30	0.15	− 0.12	0.46	0.15	0.40	− 0.11	− 0.47	− 0.44
*U. cincta*	− 0.43	− 0.38	0.46	− 0.24	0.40	0.06	0.40	− 0.56	− 0.39	− 0.44
*M. singularis*	− 0.05	0.02	0.50	0.30	0.15	0.09	0.26	− 0.47	− 0.25	− 0.48
*T. arteriosa*	0.13	0.37	− 0.29	− 0.57	− 0.32	− 0.19	− 0.12	0.65*	0.03	0.55
*P. lucia*	0.36	0.30	− 0.40	0.31	− 0.02	− 0.02	− 0.26	0.08	0.66*	0.51
*B. wilsoni*	0.02	− 0.15	0.20	− 0.27	− 0.02	0.12	− 0.34	0.38	0.00	0.13
*O. julia*	− 0.03	− 0.15	0.23	− 0.26	0.00	0.08	− 0.28	0.35	0.00	0.11

*Correlation is significant at the 0.05 level

**Correlation is significant at the 0.01 level

**Table 5:** Correlation coefficient (Pearson *r*) values of Odonata taxa as influenced by physicochemical parameters at Idi stream

Odonata	Parameters									
	Amb. temp (°C)	pH	DO (mg/L)	EC (µS/cm)	TURBIDITY (NTU)	WATER TEMP (°C)	TDS (mg/L)	BOD (mg/L)	PO_4_ (mg/L)	NO_3_ (mg/L)
*P. kersteni*	− 0.24	− 0.00	0.13	− 0.13	− 0.27	− 0.18	0.09	0.21	0.10	0.43
*P. melanicterum*	− 0.06	0.10	0.24	− 0.31	0.32	0.15	0.17	0.10	− 0.08	0.19
*P. sublacteum*	0.25	− 0.45	0.03	0.12	0.15	0.13	0.33	− 0.37	− 0.24	− 0.11
*A. victoria*	− 0.05	0.32	0.58	0.07	0.30	0.13	0.21	− 0.26	− 0.19	− 0.32
*A. subtile*	− 0.05	0.32	0.58	0.07	0.30	0.13	0.21	− 0.26	− 0.19	− 0.32
*C. pyriformosa*	− 0.05	0.32	0.58	0.07	0.30	0.13	0.21	− 0.26	− 0.19	− 0.32
*C. curta*	− 0.23	0.02	0.11	0.09	− 0.21	− 0.13	0.20	0.21	0.01	0.34
*C. glauca*	− 0.05	− 0.03	− 0.41	− 0.13	0.20	0.02	− 0.03	0.35	− 0.36	− 0.32
*E. glauca*	− 0.05	− 0.13	0.23	0.42	− 0.00	0.02	− 0.50	− 0.03	− 0.07	− 0.04
*U. cincta*	− 0.08	− 0.05	− 0.14	0.00	0.10	0.09	0.27	0.31	− 0.26	0.09
*T. arteriosa*	− 0.11	0.37	− 0.16	− 0.13	− 0.37	− 0.31	0.37	0.51	0.29	0.60*
*T. imitata*	− 0.67*	0.37	− 0.11	− 0.37	− 0.80**	− 0.93**	0.02	0.35	0.30	0.23
*P. lucia*	− 0.08	− 0.04	0.41	− 0.19	0.22	0.07	− 0.18	− 0.34	− 0.26	− 0.36
*P. portia*	− 0.05	− 0.03	0.08	0.08	− 0.01	0.09	0.32	0.13	− 0.07	0.30
*P. jucunda*	− 0.05	− 0.03	0.08	0.08	− 0.01	0.09	0.32	0.13	− 0.07	0.30
*O. julia*	− 0.00	0.01	− 0.03	0.07	0.02	0.14	0.39	0.31	− 0.03	0.38

*Correlation is significant at the 0.05 level

**Correlation is significant at the 0.01 level

**Table 6:** Correlation coefficient (Pearson *r*) values of Odonata taxa as influenced by physicochemical parameters at River Aponmu

Odonata	Parameters									
	Amb. temp (°C)	pH	DO (mg/L)	EC (µS/cm)	TURBIDITY (NTU)	WATER TEMP (°C)	TDS (mg/L)	BOD (mg/L)	PO_4_ (mg/L)	NO_3_ (mg/L)
*P. kersteni*	− 0.24	0.07	0.71*	0.63*	0.33	0.07	0.62*	− 0.44	− 0.43	− 0.44
*P. melanicterum*	− 0.31	− 0.10	0.17	− 0.12	− 0.13	− 0.02	0.27	0.06	− 0.23	− 0.16
*P. sublacteum*	− 0.21	0.17	0.07	0.14	0.31	− 0.00	0.04	− 0.14	− 0.42	− 0.25
*P. glaucum*	0.02	− 0.23	0.21	0.12	− 0.12	− 0.00	0.50	− 0.14	− 0.22	− 0.25
*P. torridum*	0.17	− 0.27	0.12	0.06	0.04	− 0.04	0.18	− 0.13	− 0.22	− 0.23
*P. sudanicum*	− 0.35	− 0.01	0.63*	0.57	0.34	− 0.00	0.41	− 0.61*	− 0.44	− 0.50
*C. glabrum*	0.81**	0.17	− 0.29	0.34	0.27	0.37	− 0.01	0.39	0.30	0.56
*A. victoria*	0.02	− 0.23	0.21	0.12	− 0.12	− 0.00	0.50	− 0.14	− 0.22	− 0.25
*A. subtile*	− 0.15	0.07	0.12	0.16	0.32	− 0.02	0.11	− 0.19	− 0.49	− 0.34
*A. vaginale*	− 0.14	− 0.05	0.16	0.10	− 0.20	0.03	0.48	− 0.06	− 0.06	− 0.11
*L. pinheyi*	0.17	− 0.05	− 0.42	0.11	− 0.12	0.37	− 0.27	0.09	0.41	0.01
*L. pallidus*	− 0.05	− 0.53	0.29	0.10	0.20	− 0.00	0.19	− 0.40	− 0.44	− 0.44
*E. glauca*	0.17	− 0.27	0.12	0.06	0.04	− 0.04	0.18	− 0.13	− 0.22	− 0.23
*M. singularis*	− 0.14	0.28	0.48	0.57	0.19	− 0.04	0.42	− 0.36	− 0.22	− 0.29
*C. curta*	− 0.14	0.28	0.48	0.57	0.19	− 0.04	0.42	− 0.36	− 0.22	− 0.29
*C. glauca*	− 0.41	− 0.42	− 0.07	− 0.26	0.40	0.08	− 0.27	− 0.23	− 0.59	− 0.34
*P. iridipennis*	0.10	− 0.29	0.26	0.24	0.59	− 0.06	0.39	− 0.31	− 0.31	− 0.36
*P. camerunensis*	− 0.46	− 0.60	0.39	0.08	0.35	0.08	0.04	− 0.59	− 0.49	− 0.48
*T. arteriosa*	0.01	0.49	− 0.23	− 0.57	− 0.03	− 0.09	− 0.58	0.52	0.44	0.47
*T. imitata*	0.49	0.50	− 0.40	− 0.19	− 0.42	− 0.38	− 0.30	0.47	0.49	0.74**
*T. furva*	− 0.14	0.50	− 0.24	− 0.59	− 0.84**	− 0.88**	− 0.39	0.24	0.36	0.44
*T. aconita*	− 0.14	− 0.38	0.07	0.13	− 0.12	− 0.04	0.40	− 0.36	− 0.12	− 0.23
*P. deceptor*	− 0.14	0.50	− 0.24	− 0.59	− 0.84**	− 0.88**	− 0.39	0.24	0.36	0.44
*P. lucia*	0.69*	− 0.01	− 0.23	0.38	0.20	0.32	0.17	0.19	0.22	0.41
*O. stemmale*	− 0.05	0.50	0.01	0.55	− 0.18	− 0.27	− 0.52	0.56	0.39	0.43
*O. guineense*	0.02	− 0.23	0.21	0.12	− 0.12	− 0.00	0.50	− 0.14	− 0.22	− 0.25
*O. chrysostigma*	− 0.01	− 0.49	0.13	0.15	− 0.08	− 0.06	0.46	− 0.39	− 0.24	− 0.34
*O. julia*	− 0.15	− 0.03	− 0.39	− 0.41	0.26	0.07	− 0.38	0.15	− 0.30	− 0.02
*B. wilsoni*	− 0.07	0.19	0.17	− 0.24	0.05	0.12	− 0.20	0.43	0.23	0.15
*P. genei*	− 0.16	0.00	− 0.42	− 0.55	0.25	− 0.10	− 0.43	0.19	− 0.32	− 0.00

*Correlation is significant at the 0.05 level

**Correlation is significant at the 0.01 level

## Discussion

Most species of Odonata (Dragonflies and Damselflies) collected in this study are common in most freshwater bodies in West Africa (Adu et al. 2014). A total of 906 individuals of dragonflies and damselflies were sampled at three sites along the course of River Aponmu. All the specimens collected were identified to lowest taxonomic level and are distributed in seven (7) families. The Libellulids which are widely known for their tolerance for disturbed habitat (Vick 2003) is the most dominant family, it accounted for 44% of all the specimens collected during the study period. According to Pilgrim and von Dohlen (2008), members of this family are widespread as about 1000 species belonging to the family have been recorded and found all over the globe. *Trithemis arteriosa* was the most dominant species and was found to be abundant at river Aponmu and also represented at the two other sites. A total of 225 individuals of the species were collected. A larger percentage of the total collected was collected at River Aponmu reaches. This is in agreement with the earlier documented information that *T. arteriosa* is usually found in slow-flowing or still water habitats (Samways 2008; Boudot et al. 2013; Dijkstra and Clausnitzer 2014). Damn et al. (2010) attributed their dominance and occurrence at variety of habitats to the fact that they belong to red-vein groups which are known for their tolerance to disturbed habitats and good dispersal ability. Many other Libellulids such as *Orthetrum Julia* recorded have been found all across West Africa (Clausnitzer and Dijkstra 2005; Adu and Ogbogu 2011). Coenagrionidae (42%) is next in terms of dominance in this study. Coenagrionids had been earlier reported by Kemabonta et al. (2016) to be good colonizers of disturbed environment. *Pseudagrion* which is the largest genus in the family with about one hundred species (Dijkstra 2003) accounted for a large number of species that represented the individuals collected in the family. *Pseudagrion Kersteni* (183) is the most abundant coenagrionid at the three study sites and they had been known to tolerate pollution and colonizer of disturbed environment. This is similar to the findings of Adu et al. (2016b), where *P. Kersteni* was found to be the most abundant Coenagrionidae at Alatori stream. Its abundance could depict that the water body is somewhat polluted. Other species of Pseudagrion present are quite few, they include *Pseudagrion melanicterum P. hamoni, P. sublacteum,* and *P. sudanicum*. *Pseudagrion* species are widespread across West and East Africa (Clausnitzer and Dijkstra 2005).

Some stenotopic damselflies belonging to Chlorocyphidae and Calopterygidae were recorded at Alaasin and Idi stream. These include *Chlorocypha Curta, Sapho Ciliata,* and *Umma cincta*. These families (Chlorocyphidae and Calopterygidae) are known for their narrow niche, sensitivity to environmental disturbance, and love for shaded streams (Subramanian 2005; Adu et al. 2016b). This explains the occurrence of more Chlorocyphidae and Calopterygidae at Alaasin and Idi which have some shaded parts.

Only one species (*Paragomphus genei*) of the family Gomphidae was accounted for during the study period. This family is known to tolerate pollution, and members of this family rarely occur in large numbers (Acquah-Lamptey et al. 2013). This further buttresses its rarity at river Aponmu which is an open river and experiences a high level of human disturbance.

Diversity and species richness in the study area indicates a slightly stable condition (Table 3). The range of values obtained for Simpson Dominance index in the three stations was high which depicts a stable community (Dash 2001). Furthermore, the occurrence frequency of *T. arteriosa* at Aponmu is very high when compared to the occurrence frequency of other species. In comparing the community structure of the study sites, Alaasin revealed a fairly good level of diversity of species and also a high level of distribution. Idi presented a poor diversity of species but fairly good level of evenness while Aponmu had the highest level of diversity in this study area but the species present are poorly distributed.

Temperature is a major parameter known to affect the quantity of dissolved oxygen (DO) in freshwater bodies. It has been reported earlier that increased Temperature usually results in low concentration of dissolved oxygen and it also influences the rate of metabolism of aquatic insects (Corbet 2004). The mean temperature values recorded in this study fall within the optimal range for tropical freshwaters. The temperature obtained at the sites shows an inverse relation with the DO values obtained which agrees with the findings of Popoola and Otalekor (2011). The change in temperature of the water body may be due to the intensity of Sunlight, time of sampling, and the environment near the water body. The two most dominant families (Libellulidae and Coenagrionidae) collected in this study are known for their adaptation to warm environment and this corroborates the findings of Suhling et al. (2015) and Kemabonta et al. (2020).

The low level of DO recorded in all the sites which is an indication of deterioration of the water quality could be due to the presence of nutrients in the water body as a result of anthropogenic activity. The pH of water influences the physiological functions of aquatic biota (Cummings et al. 2019). The pH values obtained in this study show that the pH of the water at all sites is almost neutral. Although pH has been revealed to have little or no effect on Odonata assemblage as Odonata have a wide tolerance range for pH and they are affected mostly by their habitat structure than pH (Cannings and Cannings 1994; Corbet 2004). Electrical conductivity and TDS values indicated a significant difference at river Aponmu (Table 4) which suggests a close relationship between EC and TDS since salts dissolved in water will increase the electrical conductivity of such water. This is similar to the documentation of Prommi and Payakka (2015) that increase in conductivity is an indication of dissolved ion.

## Conclusions

Freshwater quality plays a vital role in distribution, abundance, and diversity of aquatic insects, particularly Odonata. This study revealed that River Aponmu is somewhat polluted based on the similar trend in species assemblages recorded at the selected study sites. The abundance of pollution tolerant species and few stenotopic species evidenced that the water maybe experiencing a level of human disturbance at the period the research was carried out. Efforts should therefore be taken to reduce pollution in order to preserve the diversity of these insects.

## Data Availability

All analyzed data involved in this study are included in this manuscript.

## Abbreviation

PAST: Paleontological Statistics software
